# Wip1 phosphatase: between p53 and MAPK kinases pathways

**DOI:** 10.18632/oncotarget.7325

**Published:** 2016-02-11

**Authors:** Anastasia R. Goloudina, Elena Y. Kochetkova, Tatyana V. Pospelova, Oleg N. Demidov

**Affiliations:** ^1^ INSERM UMR 866, University of Burgundy, Dijon, France; ^2^ Institute of Cytology, RAS, St. Petersburg, Russia

**Keywords:** tumor suppressor, chemotherapy, phosphatase, p53

## Abstract

Cells undergoing oncogenic transformation frequently inactivate tumor suppressor pathways that could prevent their uncontrolled growth. Among those pathways p53 and p38MAPK pathways play a critical role in regulation of cell cycle, senescence and cell death in response to activation of oncogenes, stress and DNA damage. Consequently, these two pathways are important in determining the sensitivity of tumor cells to anti-cancer treatment. Wild type p53-induced phosphatase, Wip1, is involved in governance of both pathways. Recently, strategies directed to manipulation with Wip1 activity proposed to advance current day anticancer treatment and novel chemical compounds synthesized to improve specificity of manipulation with Wip1 activity. Here we reviewed the history of Wip1 studies in vitro and in vivo, in genetically modified animal models that support Wip1 role in tumorigenesis through regulation of p53 and p38MAPK pathways. Based on our knowledge we propose several recommendations for future more accurate studies of Wip1 interactions with other pathways involved in tumorigenesis using recently developed tools and for adoption of Wip1 manipulation strategies in anti-cancer therapy.

The PP2C delta phosphatase Wip1 plays an important role in normal homeostasis and pathogenesis of several human diseases [1]. These features are mainly connected with Wip1 ability to regulate signaling in MAPK kinases pathway p53 network and DNA damage response (DDR). Recently published data on the genetic polymorphism in Wip1 gene PPM1D [2] and the presence of genetic amplification and mutations of this gene in cancer [3] additionally confirmed Wip1 importance for tumorigenesis, making even more attractive an idea of Wip1 as potential oncotarget for developing new protocols in anti-cancer therapy [4]. In presented review, we will try to sort out facts from more than fifteen-year old history of Wip1 research pointing to possible benefits and side effects of manipulation with Wip1 levels and activity.

Wip1 was first found as p53 target gene and named, Wip1 (wild-type p53-induced phosphatase), in the laboratory of Ettore Appella [5]. Interestingly, that in this first report authors presented evidences that Wip1 overexpression negatively regulates clonal survival in two tumor cell lines, T98G and Saos2. However, further studies by several groups identified targets of Wip1-dependent dephosphorylation and proved that Wip1 revealed features of mild oncogene rather than tumor suppressor. The initial controversy as we currently understand was due to the p53-negative status of both cell lines, T98G and Saos2, used in the original study. The p53 status, with active or compromised p53 pathway, is crucial for prediction of effects from manipulation with Wip1 activity or levels.

Below, we compare effects of increasing Wip1 activity versus Wip1 inhibition focusing on data obtained mainly in experiments with genetically modified mice.

## WIP1 DELETION AND INHIBITION

### Wip1, DDR and p53 pathway

**Figure 1:**
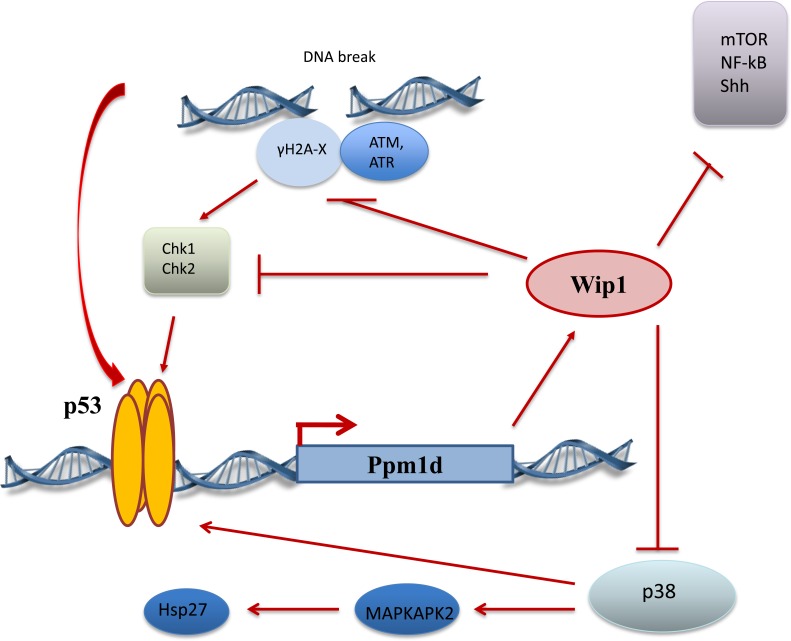
Wip1 regulation and targets

Currently, Wip1 is considered as a bona fide negative regulator of p53 pathway. p53 itself is the most potent transcriptional activator regulating transcription of Wip1 gene, PPM1D, compared to other identified Wip1 regulating transcriptional factors, such as NFkB [6], CREB [7], ER [8], c-Jun [9] and others. The cascade of events after DNA damage leads to p53 activation, subsequent binding Wip1 promoter and turning ON Wip1 transcription that resulted in elevated levels of Wip1 protein. Wip1 directly dephosphorylates p53 on Ser 15 [10], hence reducing, p53 activity and levels, but more importantly in our opinion Wip1 down regulates the whole system of positive signaling to p53 from damaged DNA. ATM [11], Chk1[10], Chk2 [12], γH2AX [13, 14, 15, 16] proteins, which are responsible for sensing DNA damage and activating DDR, were shown to be targeted and negatively regulated by Wip1 (Figure 1). It was also shown by the group of L. Donehower that Wip1 could potentiate MDM2 function to further reduce p53 levels [17].

This feedback mechanism of p53 - Wip1 relations is thought to be necessary for cell recovery after DNA damage and some forms of stress. The fine-tuning of p53 response is also vital for the prevention of cell death or senescence especially during development and in stem cell homeostasis.

p53 and members of DDR system play a significant role in tumor suppression and many anti-cancer strategies are based on their activation. In agreement with this fact, more active p53 pathway in mice with genetic deletion of Wip1 significantly attenuated tumorigenesis in several tumor models, APC (Min) intestinal tumorigenesis [18], c-myc induced lymphoma [10] and others. In these models the mediating role of p53 was confirmed by reversing tumor suppressive phenotype of Wip1−/− mice by deletion of p53. At the same time, Wip1 deletion was unable to prevent lymphomas and sarcomas associated with p53−/− phenotype. The negative consequences of Wip1 deletion such as premature aging [19], lymphopenia [20] also could be linked to the abnormal regulation of p53 pathway and in many cases cured by p53 inhibition.

These facts lead to the conclusion that Wip1 inhibition strategy could sensitize p53 to upstream signaling, including DDR and oncogenic stress, and thus prevent tumorigenesis. However, one has to take into account possible side effects in normal tissues with elevated activity of DDR, high proliferation pace and therefore highly sensitive to DNA damage.

Interestingly, Wip1 deletion protected mice from mammary tumorigenesis [21]. From three models of breast cancer studied by Bulavin and others Wip1 deletion attenuated mammary gland tumorigenesis only in MMTV -Erbb2 and MMTV-HRAS1 mice, but not in MMTV-WNT1 mice [22]. The authors did not study deeply effects of p53 deletion in mammary gland on the Wip1-dependent tumor suppression, but linked the observed effect to another direct target of Wip1 - p38 MAPK.

## WIP1, P38 AND OTHERS

Takekawa et al 2000 found that Wip1 directly binds and inactivates p38 MAPK by dephosphorylation of Thr182 activatory site under stress conditions such as H2O2 treatment or UV-irradiation [23]. The activation of p38 can parallel with the simultaneous activation of p53 and plays direct role in positive posttranslational regulation of p53. Despite possible crosstalk between MAPK pathway and p53 pathway we foresee that it will be important to distinguish p53-dependent and p38-dependent effects of Wip1 deletion and/or activation. In the study mentioned above (Bulavin and colleagues [22]) the absence of inhibitory effect of Wip1 on p38 leads to elevated levels of two products of Cdkn2a gene, p16 and p14^arf^, which were responsible for tumor suppression in mammary gland epithelium. Pietersen's group followed up study on mammary gland development in Wip1−/− mice deepened our knowledge showing that Wip1 effect is restricted to the estrogen receptor positive cells of luminal layer of mouse mammary gland epithelium. This finding correlates with data from patients with breast cancer. It has been shown that the amplification of and mutations in the PPM1D gene occurred mainly in the luminal subtype of human breast cancer [24]. This group also showed that Wip1 could regulate activity of Stat5 transcriptional factor and another member of MAPK family - ERK kinase.

**Figure 2:**
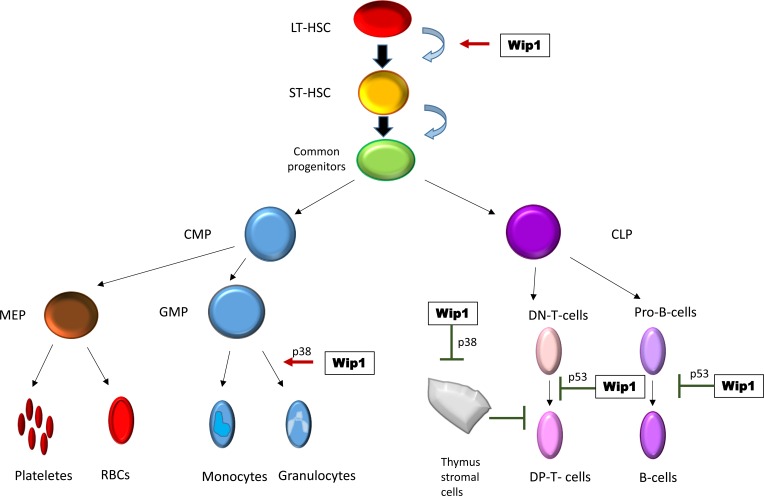
Wip1 in regulation of hematopoiesis

In addition to intrinsic interplay between Wip1, p53, p38 and other factors in tumors cells, tumorigenesis could be affected by changes in immune system of Wip1−/− mice (Figure 2).

Though defect in T-cell and B-cell maturation was attributed largely to the function of p53 with no considered as p53-dependent effect with no involvement of p38 [19, 23], the increase in granulocyte compartment of hematopoietic system was linked to p38 proficiency in Wip1−/− mice [25]. This disturbance in immune system, regulation of stromal cells [26], involvement of p38 pathway in cytokine secretion and described proinflammatory phenotype of Wip1 −/− mice [27, 28] allow us to hypothesize that tumorigenesis could be affected not only at the level of tumor cells themselves, but by tumor microenvironment as well. This hypothesis requires new approaches and tools such as conditional deletion of Wip1 in specific cells or tissues. Single reports on Wip1 involvement in regulation of other MAPK kinases: ERK [29] and JNK [8, 30], NFkB [31, 32] pathway additionally motivate community to conduct such experiments.

Unfortunately, at the present moment the data on genetic deletion of Wip1 in mice can not be fully supported by studies which used Wip1 inhibitors instead of genetic deletion or siRNA/shRNA depletion shRNA depletion due to reported off-target effects of current day Wip1 inhibitors [33]. The careful adjustment of inhibitor concentration could be performed in experiments with cell lines in culture, but more complex pharmacological aspects of in vivo experiments makes interpretation of data obtained in such studies with animals a difficult task. Recently, two new promising Wip1 inhibitors reported to have a high specificity towards Wip1 inhibition. They can potentiate cytotoxic effects of chemotherapeutic agents in wild type p53 tumor cells, though their potential side effects and usefulness for anti-cancer therapy await further studies in vivo [34, 35, 36].

As an additional remark to the data obtained from knockout animals, we would like to mention, that in all experiments we conducted on mice with genetically modified Wip1 no haploinsufficiency was observed. It seems that expression of Wip1 only from one allele is sufficient for proper function and does not disturb phenotype at least in normal development and tumorigenesis. On the other hand, it is important to mention that Wip1 deletion (Wip1−/−) in heterozygous p53+/− animals applies selective pressure on p53 pathway during tumorigenesis and frequently leads to the complete loss of p53 in tumors [11].

## WIP1 OVEREXPRESSION

Recently, significant number of studies published on Wip1 overexpression in various tumors that correlates or not with status of positive p53 status in these tumors. Some studies emphasize on very important fact that Wip1 overexpression predominantly observed in tumors without p53 mutations highlighting a fact that Wip1 overexpression gives no advantages to the tumors without functional p53 tumor suppressor pathway. The critical assessment of such studies indicated that presented data especially on protein Wip1 levels could be sometime confusing and contaminating informational landscape. Different poorly described and verified anti-Wip1 antibodies from different companies were used in these studies frequently showing figures with cytosolic localization of mainly nuclear Wip1 protein. The new verified anti-Wip1 antibodies recently announced by the companies with long-time expertise in antibodies production will allow verification and refining of previously published data.

Currently, two mouse models with Wip1 overexpression were reported: ubiquitous Wip1 transgenic mouse, Ub-Wip1 [37], and mammary gland specific Wip1 transgenic mouse, MMTV-Wip1 [38].

Despite the fact that Wip1 overexpression was reported in many human cancers, and correlated with the more aggressive stage and poor prognosis, no spontaneous tumor appearance was observed in mice with Wip1 overexpression. One of the explanations to this phenomenon can be that the life span of mice is not sufficient to accumulate secondary to Wip1 mutations required for tumor formation. Thus, it can be assumed that overexpression of Wip1 has potentiating effect but not sufficient to trigger tumorigenesis. In other words, it only sets up the stage for cancer-driving mutations.

In the breast cancer animal model Wip1 overexpression significantly accelerated mammary gland tumor formation MMTV- Erbb2/MMTV-Wip1 mice. The oncogenic effect was not dependent on p53 status. No differences was observed in MMTV- Erbb2/MMTV-Wip1/p53−/− mice. On the contrary, introduction of an active copy of the MKK6 (MMTV-MKK6*) into the mammary gland epithelium minimized the pro-oncogenic effect of Wip1 overexpression [39].

Interestingly, that the initial observation by Fiscella et al 1998 that Wip1 overexpression could have some tumorsupressive effect was confirmed later [41], but it is true only for tumors with negative p53 status.

For some cell lines, e.g. MCF7, with with amplification of the PPM1D chromosomal locus or some patient derived tumor cell explants with overexpressed Wip1, the uncontrolled cell cycling and extended resistance to apoptosis was supported by the fact of negative influence of elevated Wip1 levels on activity of p53 pathway [40].

The opposite effect was observed in tumor cell lines with p53 negative status.

p53 is one of the most important tumor suppressors regulating initial steps of oncogenic transformation, tumor growth and response to anti-cancer treatments. It is also one of the most frequently mutated genes in cancer. In some cancers e,g, large intestine, p53 mutations occur in majority of the cases according to Welcome Trust Sanger Institute catalogue of somatic mutations in cancer. The consequences of different types of mutations in p53 gene can vary. They could either mildly affect p53 functions or culminate in lost-of- function and/or gain of alternative functions. Chromosomal deletion or mutations, compromising p53 DNA binding, abrogate p53 transcriptional functions. Hence, the initiation of apoptotic program in response to DNA damage inducing agents can be abolished due to insufficient increase in expression of pro-apoptotic genes and lack of suppression of anti-apoptotic proteins. The unchanged in response to therapeutic stress pro-/ anti-apoptotic ratio can be responsible for drug resistance in tumors. For example, in our model osteosarcoma cell line Saos2 mutation in p53 gene allows cells to tolerate well high doses of chemotherapeutic drugs e.g. cisplatin, that are usually toxic for cells with preserved p53 functions. The absence of p53 nullify positive anti-tumor effect of Wip1 depletion/inhibiton observed in cells with p53 functions are still intact [39].

On the contrary, after disappearance of p53 as the main direct and indirect substrate for Wip1, new functions of Wip1 and its new substrates can be more easily revealed and become more important for cellular physiology. We found that inducible overexpression of Wip1 in Saos2 compensated tumor cells response to chemotherapy for the loss of p53. Tumor cells became more sensitive to cisplatin and underwent caspase-3 dependent apoptosis due to restored shift of Bax/Bcl-XL ratio towards pro-apototic protein. In the absence of p53 Wip1 regulated transcriptional factor Runx2 binding to specific RUNT elements in Bax promoter and activation of Bax transcription by dephosphorylation of critical inhibitory sites in Runx2 molecule [41]. Furthermore, Wip1 suppressed anti-apoptotic BCL-Xl expression by interfering with NFkB pathway [42].

The peculiar fact is that to increase efficiency of DNA-damaging drug, such as cisplatin, in p53 negative cells you do not need the increase in DDR. Normally, double strand breaks in DNA induce cascade of signaling events including phosphorylation of histone γH2AX on Ser-139. This phosphorylation is a marker an early initiation point of DDR resulted in cell cycle arrest or cell death. The DNA damage induced γH2AX phosphorylation was reduced in Wip1 overexpressing cells as Wip1 targets the p-Ser 139 site. At the same time these cells with lower phospho-γH2AX levels were more responsive to cisplatin than control Saos2 cells with high phospho-γH2AX levels. This observation accentuate the fact that phospho-γH2AX levels as a marker of DDR, does not always correlate with toxicity of chemotherapeutic drug.

This phenomena is important so as in normal tissues hyper phosphorylation of γH2AX could induced extensive signaling in p53 pathway resulted in cell death and damage to normal tissues. Wip1 is capable to decrease the toxic DNA damage signaling from chemotherapeutic agents to p53 in normal tissues, so as p53 functions preserved in all other organs and tissues except tumors with p53 mutations. Prevention of cell death in highly sensitive to DNA damage tissues with high rate of proliferation such as intestinal epithelium, testes, and hematopoietic cells could reduce side effects of anti-cancer therapy that together with increased sensitivity of p53-negative tumor cells will widen therapeutic window and allow selection of optimal dosage for anti-cancer drug.

Currently, Wip1 is established and well confirmed by significant body of publications as a regulator of two main pathways, DDR-p53 and MAPK-kinases. Recently announced involvement of Wip1 phosphatase in regulation of other pathways, such as Stats, NFkB [43], mTOR, Sonic Hedgehog [44] and Notch signaling requires additional clarification so as non-specific Wip1 inhibitor (CCT007093) was used in many studies and these observation could be purely correlative.

## WIP1, MTOR AND AUTOPHAGY

Recent consideration of Wip1 as a potential oncogene allows to develop hypotheses about links between Wip1 activity and several processes, crucial for carcinogenesis, including autophagy. Autophagy (macroautophagy) is an evolutionarily conserved degradation process, during which proteins or damaged organelles (cargo) are isolated into double-membrane vesicles and digested as a result of fusion with lysosomes [45, 46]. Autophagy is an essential process that occurs throughout all cell types, supporting normal homeostasis as well as helping to cope with stress conditions and carcinogenesis [47, 48]. Role of autophagy for cancer cells is dual, depending on stage of transformation and cell type. Autophagy is inhibited by multiple oncogene products, including PI3K, AKT, BCL-2 and mutant p53, and inhibition of autophagy is a poten­tially oncogenic event [49]. However whereas at the stage of solid tumor autophagy can serve as rescue pathway for cancer cells that face lack of oxygen and nutrients, thus inhibition of autophagy at this stage can lead to elimination of malignant cells. Being an important process for cancer cells, autophagy is studied as a potential target for cancer therapy.

A suggestion can be made that Wip1 phosphatase is somehow involved in autophagy regulation. One may suppose that the links are in DNA damage response regulation. Autophagic process seems to be in a tight interplay with DDR [50, 51], some DDR participants being shown to contribute to autophagy regulation: ATM [52], PARP-1 [53] p53 [54, 55]. Autophagy, in turn, was shown to contribute to maintaining genome stability [56, 57]. These authors have shown that loss of core autophagic protein Beclin 1 functions increase genome abnormalities. Autophagic protein FIP200 deficiency turned out to lead to lag in DNA repair [58]. However, despite the facts of significance of autophagy for genome stability, connecting Wip1 with autophagy through Wip1 interactions with DDR participants still needs experimental proves.

A more promissing candidate for a link between Wip1 and autophagy is mTOR kinase. mTOR is known as a key regulator of cellular senescence [59, 60, 61, 62], stress response and cell death [63, 64, 65]. mTOR exists in two complexes - mTORC1 and mTORC2, mTORC1 being a negative regulator of autophagy [66, 67]. First, Wip1 was linked to autophagy in the study conducted by Le Guezennec et al [68]. They have shown involvement Wip1 in regulation of autophagy in mice during conversion of macrophages into foam cells in atherosclerosis. Authors suggested involvement of ATM and mTOR in this process. Previously, links between Wip1 and mTOR signaling were only to be assumed, built on the fact that Wip1 dephosphorylates several proteins that regulate mTOR activity directly or indirectly. These include p53, which affects mTOR activity and autophagy [69, 70]. p53 binds to the promoters of genes, the protein products of which regulate autophagy, including upstream regula­tors, such as the β1, β2 and γ-subunits of AMP-activated protein kinase (AMPK), several regulators of the AMPK and mTOR1 complexes, sestrin 1 and sestrin 2 proteins, connect genotoxic stress and mTOR signaling [71]. Recently catalogue of autophagy-relevant genes induced by p53 was published [72]. In addition to *Ulk1*, authors identified a set of autophagy genes, including *Atg2b, Atg4a, Atg4c, Atg7, Atg10, Tmem49/Vmp1, Ulk2*, and *Uvrag*, as p53-responsive. Furthermore, it was as previously found, lysosomal protein also bound by p53 [73].

Then, another Wip1 target p38 was shown to participate in mTOR signaling activity regulation, for example, through interactions with mTORC1 component Raptor [74] or with Rag GTPases that regulate mTORC1 in response to amino acid modulation and stress [75, 76]. Also, p38 is involved into the regulation of autophagy. It was shown that p38 suppressed starvation-induced autophagy by regulating autophagic protein mAtg9 [77]. In the other hand, modulation of p38 activity was shown to affect LC3 conversion from LC3 I to LC3 II, which is a necessary event for the occurrence of autophagy [78, 79]. On the whole, it appears that influence of p38 on autophagy depends on cell type and conditions. However, the affect of p38 on autophagy through mTOR signaling is still to investigate.

Importantly, recently it was shown that Wip1 can directly dephosphorylate mTOR [80]. Authors have found that Wip1−/− mice exhibit enhanced liver regeneration after partial hepatectomy, characterized by increased levels of phosphorylated mTOR (Ser2448, Ser2481, Ser2159), as well as it's phosphorylated targets p70S6K and S6. Enhanced liver regeneration and mTOR activity were linked with increased proliferation. Nevertheless, mTOR kinase is not only involved in proliferation, but also in cellular senescence, stress response, cell death, negative autophagy regulation. Inhibition or enhancement of these processes is an important event for carcinogenesis, these facts allow to suggest a new signaling network between Wip1 and mTOR, that can be considered as a potential target for modulation in anticancer therapy.

## CONCLUSIONS AND PERSPECTIVES

1. Wip1 is a potential oncotarget and strategies directed to manipulation with its levels/activity could benefit modern day anti-cancer therapeutics. All current and future data on Wip1 polymorphism/mutations in tumors or manipulations with Wip1 activity/levels should take into account p53 status, so as p53 gene is one most frequently mutated genes in tumors and its status affects Wip1 expression and actions. All previously accumulated data should be re-analyzed carefully taking this note under consideration.

2. Wip1 levels/activity could be important diagnostic and prognostic tool in oncology. The clinical development of this tool required more specific anti-Wip1 antibodies, analysis of nuclear versus cytoplasm localization of Wip1, and taking into the account regulators of Wip1 expression: p53, DNA repair genes, MAPK, NFkB status of the tumor.

3. In tumors with preserved wild type p53 pathway Wip1 inhibition increases tumor suppressive effect of p53, but this strategy should take into account that Wip1 inhibition in normal tissues and their stem cell compartment significantly increases sensitivity to current day anti-cancer therapy. In this type of tumor Wip1 is a better alternative to MDM2 inhibitors, because it does not affect p53 levels directly and does not immediately increase apoptosis in normal tissues per se, but rather mildly affects sensitivity of p53 for upstream signaling during DDR.

4. In more than half of all tumors p53 is mutated and Wip1 inhibition is ineffective. On contrary, temporary Wip1 activation could increases sensitivity to chemotherapeutic drugs and decreases side effects of DDR in normal tissues. The current day oncological practice could benefit from this strategy of Wip1 activation/p53 downregulation synthetic sensitivity to chemotherapeutic drugs.

5. Wip1 is a potent anti-aging, anti-inflammatory and neuromodulator agent, but these type of studies should take into account possible pro-tumorigenic effect of Wip1 activation.

6. The perspective of further studies is understanding of Wip1 interplay with multiple pathways on the cellular level (nuclear and cytoplasm) and whole organism level (secretion and immune system), finding new regulators and targets of Wip1, as potential therapeutic tool.

Taking into account information that was written above the most urgent task in the field is creation of specific anti-Wip1 assays as diagnostic tool and developing new bioavailable inhibitors/activators of Wip1 as promising therapeutics for tumor prevention and anti-cancer treatment that could significantly improve success rate in the treatment of oncological diseases.
